# Molecular Extraction and Release of an Ionic Liquid Microdroplet With Dynamic Tunability Under Optical Trapping

**DOI:** 10.1002/cphc.70445

**Published:** 2026-06-11

**Authors:** Kosuke Nakatsu, Rai Kobayashi, Moe Akazawa, Yasuyuki Tsuboi, Ken‐ichi Yuyama

**Affiliations:** ^1^ Department of Chemistry Graduate School of Science Osaka Metropolitan University Osaka‐shi Japan

**Keywords:** fluorescence spectroscopy, liquid–liquid phase separation, lower critical solution temperature, optical tweezers, Raman spectroscopy

## Abstract

Ionic liquids (ILs) exhibiting temperature‐responsive phase separation offer promising opportunities for microscale extraction when combined with optical tweezers. Here, we induce local phase separation to generate a single IL‐rich microdroplet under optical trapping and investigate the dynamics of molecular extraction into, and release from, the droplet using microspectroscopic analysis. At the high laser power, the microdroplet grows and becomes concentrated, thereby promoting molecular extraction. Upon reducing the laser power, the droplet shrinks while maintaining a high IL concentration. During this process, a dense domain forms around the shrinking droplet, accelerating molecular release from the droplet. Furthermore, we find that the local microenvironment experienced by the extracted molecules is governed by the concentration balance between the IL and the solute molecules. These findings elucidate the key factors controlling molecular extraction and release in an optically manipulated IL microdroplet and provide insights into the design of a new single‐droplet‐based approach for microanalysis.

## Graphical abstract

We produce a single microdroplet by inducing local phase separation of a temperature‐responsive ionic liquid aqueous solution under optical trapping conditions. As the droplet grows, molecules in the surrounding solution are extracted to the droplet. Upon droplet shrinkage, these molecules are released from the droplet. The molecular release process is promoted with a dense domain surrounding the droplet.

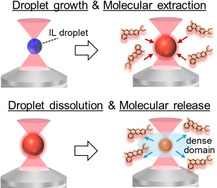

## 1 Introduction

Ionic liquids (ILs) have attracted considerable attention as novel solvents exhibiting physicochemical properties that are much different from those of conventional molecular liquids. Their properties can be precisely tuned by varying constituent cations and anions; therefore, ILs are often regarded as “designer solvents.” Due to this tunability, ILs have found applications across a wide range of fields, including electrochemistry, nanotechnology, analytical chemistry, and extraction processes [1, 2, 3, 4, 5]. Among ILs developed to date, some exhibit temperature‐responsive liquid–liquid phase separation (LLPS) behavior [6, 7]. These ILs have a lower critical solution temperature (LCST) in aqueous mixtures. Upon heating above the LCST, the homogeneous mixture separates into IL‐rich and water‐rich phases. The single‐phase state is restored with cooling. Such reversible phase separation offers promising opportunities for extraction and separation of diverse molecules. Indeed, Kohno et al. demonstrated efficient extraction of dyes and proteins into the IL‐rich phase through LCST‐type LLPS in IL/water mixtures [8, 9].

In such LLPS, the interfacial area of the IL phase plays a crucial role in extraction efficiency. As an IL phase decreases in size, the extraction efficiency increases. Therefore, it is a highly promising strategy to generate a single IL microdroplet through localized phase separation, which is for the efficient extraction of target molecules. Optical tweezers serve as a powerful tool to induce such localized phase separation in various systems, including temperature‐responsive solutions [10, 11, 12, 13, 14]. Irradiation with a tightly focused 1064 nm laser beam generates not only an optical trapping potential but also a local photothermal effect [15]. The latter arises from the absorption of 1064 nm photons through overtone of the O–H vibrational modes of water, causing a temperature rise near the focal point. When the local temperature reaches close to the LCST, phase separation is induced to form condensates, which are subsequently confined within the optical potential well. These condensates ultimately coalesce into a single microdroplet, typically 10–30 µm in diameter. This single‐droplet formation was first demonstrated in the polymer aqueous solution and has been extended to other LCST‐type LLPS systems, including aqueous alcohol, polymer, protein, and IL solutions [16, 17, 18, 19, 20, 21, 22, 23].

For ILs, we previously demonstrated single‐droplet formation in aqueous solutions of tetrabutylphosphonium 2,4‐dimethylbenzenesulfonate ([P_4444_]^+^[2,4‐MeSO_3_]^−^), tetrabutylphosphonium trifluoroacetate ([P_4444_]^+^[CF_3_COO]^−^), and tributyl‐*n*‐octylphosphonium bromide ([P_4448_]^+^[Br]^−^), as well as the successful extraction of dye molecules and nanoparticles into these droplets [19, 20, 21]. Although we have examined extraction behavior by microspectroscopy during droplet growth, the mechanism by which extracted molecules are released from the droplet remains unclear. In this study, we produce an IL single droplet under optical trapping conditions and investigate molecular extraction/release dynamics during the growth/dissolution processes of the single droplet, respectively. Raman and fluorescence microspectroscopy reveal that the droplet dissolution and the accompanying molecular release are strongly influenced by the surrounding solution, particularly by the presence of a dense domain around the droplet. These findings provide useful insights for optically controlling a single microdroplet and designing single‐droplet‐based microanalysis.

## 2 Experiments

The IL used in this study was [P_4448_]^+^[Br]^−^. It was dissolved in water, and the IL concentration (*C*_IL_) was adjusted to 5.0 wt%. The critical temperature of the prepared aqueous solution was ≈40°C. Single‐droplet formation was induced by tightly focusing a continuous‐wave (cw) 1064 nm laser beam into the solution using an oil‐immersion objective lens (numerical aperture: 1.45). The effective power (*P*_eff_) of the near‐infrared (NIR) laser beam was measured after the objective lens and adjusted to either 600 or 400 mW. A single IL droplet was generated at the laser focus and subsequently grew at 600 mW, whereas it shrank at 400 mW. For fluorescence analysis, Nile red (NR) was added to the initial solution at a concentration of 5.0 µmol/L. The dye molecules were extracted into an IL droplet during the growth process, while they were released from the droplet upon shrinkage. The processes of droplet formation, growth, and dissolution were examined by optical transmission and fluorescence imaging, as well as Raman and fluorescence microspectroscopy. Details of the optical setup and the sample preparation are described elsewhere [20, 21].

## 3 Results and Discussion

**FIGURE 1:**
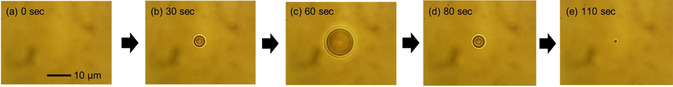
(a) Optical transmission micrographs under the irradiation of a focused NIR laser beam. The *P*_eff_ was 600 mW at 0–60 s (a–c) and 400 mW at 60–110 s (d–e).

Single‐droplet formation was clearly observed by optical transmission imaging during irradiation with the NIR laser beam (*P*_eff_ = 600 mW) at room temperature (25°C) (Figure 1). The aqueous IL solution was initially homogeneous, and no apparent changes were detected in the micrograph at the onset of irradiation (Figure 1a). After several tens of seconds of continuous irradiation, a small liquid droplet emerged at the focal point of the NIR laser. The droplet subsequently exhibited steady growth while being stably trapped at the laser focus (Figure 1b,c). Red fluorescence was observed at the focal point, which was attributed to two‐photon excitation of NR by the tightly focused NIR laser beam. When *P*_eff_ was reduced to 400 mW after 60 s, the droplet began to shrink, and its volume gradually decreased (Figure 1d,e). Throughout this dissolution process, the droplet remained stably trapped at the laser focus.

**FIGURE 2:**
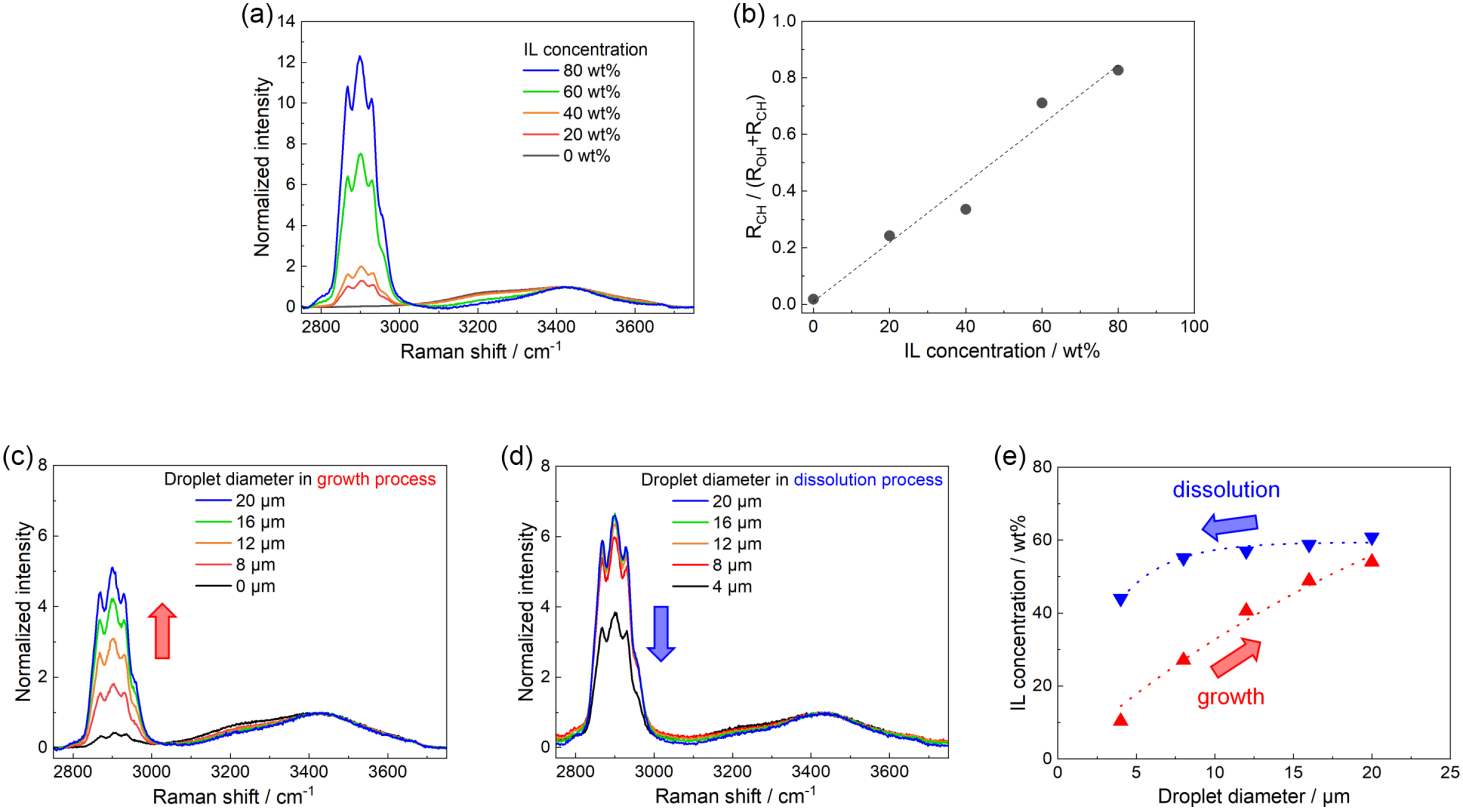
(a) Raman spectra of bulk IL aqueous solutions with varying *C*_IL_. (b) A calibration curve showing the relationship between *C*_IL_ and the Raman band area ratio [*R*_CH_/*R*_OH_ + *R*_CH_]. Raman spectra of droplets with different diameters generated by the NIR laser optical tweezing technique in the (c) growth and (d) dissolution processes. (e) Relationship between droplet diameter and *C*_IL_, using droplets generated via optical tweezing in the growth and dissolution processes. Dotted lines are fitted curves for the relationships in the respective processes. The spectra shown in (a), (c), and (d) were normalized using the peak intensities in the O–H stretching region.

To estimate *C*_IL_ within individual droplets generated under optical trapping conditions, we combined optical tweezers with Raman microspectroscopy. For the spectroscopic measurements, IL solutions without NR were employed. Initially, a calibration curve was constructed by using bulk IL/water mixtures with *C*_IL_ of 0–80 wt%. Raman spectra were recorded under steady‐state conditions (Figure 2a). Two distinct Raman bands were observed in the C–H (2800–3000 cm^−1^) and O–H (3000–3700 cm^−1^) stretching regions, corresponding to the IL and water components, respectively. The calibration curve was obtained by plotting the ratio of Raman band areas, [*R*_CH_/(*R*_OH_ + *R*_CH_)], as a function of *C*_IL_ (Figure 2b).

Single IL microdroplets of varying sizes were generated under optical trapping conditions in the 5.0 wt% aqueous IL solution, and their Raman spectra were recorded (Figure 2c). To maintain a constant droplet size during the measurements, *P*_eff_ was slightly reduced and carefully adjusted once the droplet reached the desired diameter. Raman spectra acquired were normalized to the respective peak intensities of the O–H stretching bands. As the droplet size increased, the relative intensity of the C–H stretching region became higher. From these spectra, the Raman band area ratio, [*R*_CH_/(*R*_OH_ + *R*_CH_)], was calculated, and the corresponding *C*_IL_ values were estimated with the use of the calibration curve. We found that *C*_IL_ increased almost linearly with droplet diameter, reaching ≈50–60 wt% for a droplet with a diameter of 20 µm (Figure 2e). These results demonstrate that single IL microdroplets with tunable sizes and concentrations can be controllably prepared under optical trapping conditions.

Individual droplets undergoing dissolution were also investigated in the same manner as described above. Figure 2d shows the Raman spectra of dissolving droplets with various sizes (*P*_eff_ = 400 mW). The droplet size was maintained constant during the measurements by carefully adjusting *P*_eff_ once the desired diameter was reached. The Raman band area ratios, [*R*_CH_/ (*R*_OH_ + *R*_CH_)], were calculated to estimate the corresponding *C*_IL_ values (Figure 2e). As the droplet diameter decreased from 20 to 8 µm, *C*_IL_ remained nearly constant (≈55 wt%). However, a pronounced decrease in *C*_IL_ was observed when the diameter further decreased to 4 µm. Thus, the size dependence of *C*_IL_ during dissolution differed from that observed during the growth process. When comparing droplets of the same size, the droplet undergoing dissolution has a higher *C*_IL_. This difference is discussed later.

Incidentally, the O–H stretching bands clearly changed in their shape depending on *C*_IL_ both in steady‐state bulk solutions (Figure 2a) and in growing or shrinking microdroplets (Figure 2c,d). The relative intensity around 3200 cm^−1^ became weak compared to the peak intensity as *C*_IL_ increased. It is well known that the spectral region around 3200 cm^−1^ is highly sensitive to hydrogen‐bonding network of water. The Raman signal in this range weakens when the network is disrupted, for instance, by heating or adding salts [24, 25]. In the present system, the IL disrupts hydrogen‐bonding network of solvent water molecules through strong electrostatic interactions.

NR was employed as a fluorescence probe to evaluate molecular extraction efficiency of IL droplets in the growth and dissolution processes. In addition to the extraction efficiency, NR gives us information on the surrounding environment, because the dye exhibits fluorescence that is highly sensitive to polarity of solvents (Figure S1) [26]. Its emission maximum shifts to a shorter wavelength as solvent polarity decreases. NR is often used for staining intracellular lipids [27]. Therefore, we expect that NR strongly interacts with the alkyl chains of the [P_4448_]^+^ cation owing to structural similarity with lipids and the fluorescence color vary depending on the local solvation environment within IL droplets.

**FIGURE 3:**
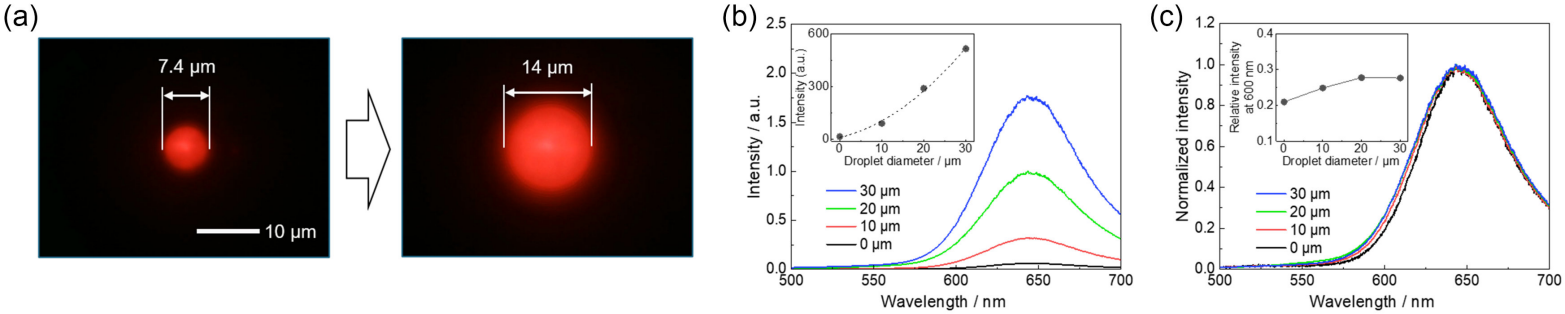
Fluorescence (a) micrographs and (b) spectra of IL droplets growing under optical trapping conditions. The inset of (b) is the integrated fluorescence intensity that was plotted against the droplet diameter. (c) Normalized fluorescence spectra. The inset presents the intensity at 600 nm against the droplet diameter.

Figure 3a shows the temporal change in the fluorescence images near the focal spot of the NIR laser beam (*P*_eff_ = 600 mW). These images were captured under wide‐field illumination using a cw 405 nm excitation laser beam. Prior to the droplet formation, the solution exhibited negligibly small fluorescence in the image due to the low NR concentration. In contrast, IL droplets exhibited bright red fluorescence, which results from NR extracted into the droplets. Fluorescence spectra were recorded to further evaluate the extraction efficiency (Figure 3b). The fluorescence band was observed at 550–700 nm, and its intensity increased across the entire wavelength range with the droplet growth (the inset of Figure 3b). This intensity enhancement is attributed to the progressive extraction of NR into the growing IL droplet. As the droplet grows, it becomes more hydrophobic due to a higher *C*_IL_, enhancing the extraction of NR.

A slight change in the spectral shape was observed during the droplet growth. Figure 3c shows the fluorescence spectra normalized to their respective peak intensities. Notably, the relative intensity in the 575–625 nm region (spectral tail) was enhanced with increasing droplet size (the inset of Figure 3c). This result suggests that the local environment of NR within the droplet becomes more hydrophobic as the droplet grows to increase *C*_IL_. Indeed, NR exhibits a clear IL‐concentration‐dependent fluorescence behavior in bulk solutions, where a distinct blue shift in emission was observed with the change in *C*_IL_ from 30 to 100 wt% (Figure S2). Note that NR exhibits thermochromic behavior [28]. However, the fluorescence spectra measured over a temperature increase of 13 K showed no noticeable spectral shift (Figure S3). Therefore, we conclude that the thermochromic effect of NR is negligibly small under the present experimental conditions.

**FIGURE 4:**
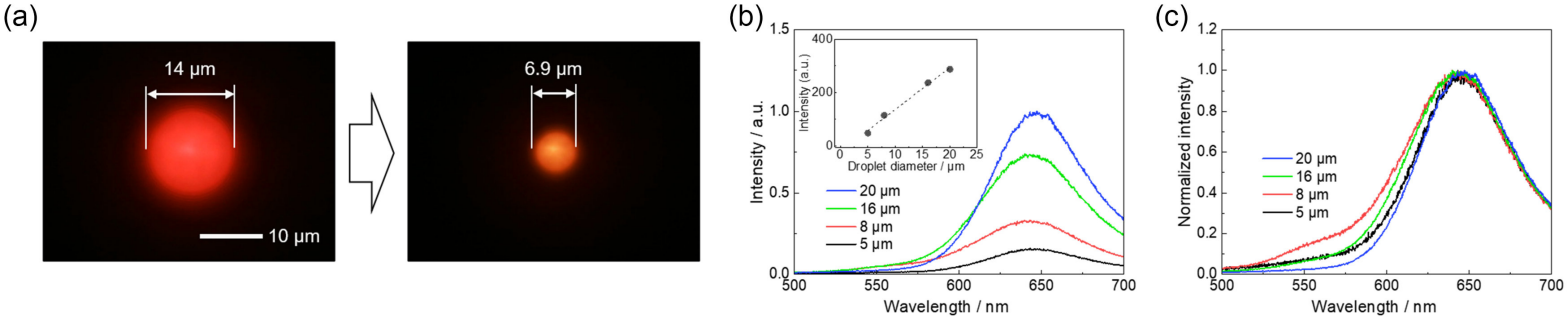
Fluorescence (a) micrographs and (b) spectra of IL droplets dissolving under optical trapping conditions. The inset of (b) is the integrated fluorescence intensity that was plotted against the droplet diameter. (c) Normalized fluorescence spectra.

The molecular release dynamics during the droplet dissolution (*P*_eff_ = 400 mW) was also investigated by fluorescence imaging/microspectroscopy. Interestingly, the fluorescence color changed from red to orange as the droplet shrank (Figure 4a). Subsequently, the color reverted to red. The orange fluorescence was not observed during droplet growth, where the droplet exhibited only red emission. To clarify the origin of this color variation, fluorescence spectra were recorded throughout the dissolution process (Figure 4b). At an initial droplet diameter of 20 µm, the fluorescence peak was located at 650 nm. As the droplet size decreased, the overall fluorescence intensity diminished (inset of Figure 4b). Notably, the spectral profile was dynamically changed during dissolution. To emphasize this change, the spectra were normalized to their respective peak intensities (Figure 4c). When the droplet diameter decreased from 20 to 8 µm, a slight blue shift of the emission peak was observed, together with the increase in the relative intensity at 525–600 nm. This spectral shape changes can account for the apparent fluorescence color variation from red to orange. However, the color evolution is opposite to that expected from the *C*_IL_ dependence of NR emission. A blue shift typically occurs with increasing *C*_IL_ concentration. In contrast, *C*_IL_ decreases during droplet shrinkage. Therefore, the results shown in Figure 4 cannot be explained solely from the viewpoint of *C*_IL_, suggesting the presence of an additional factor influencing the local solvation environment of NR.

**FIGURE 5:**
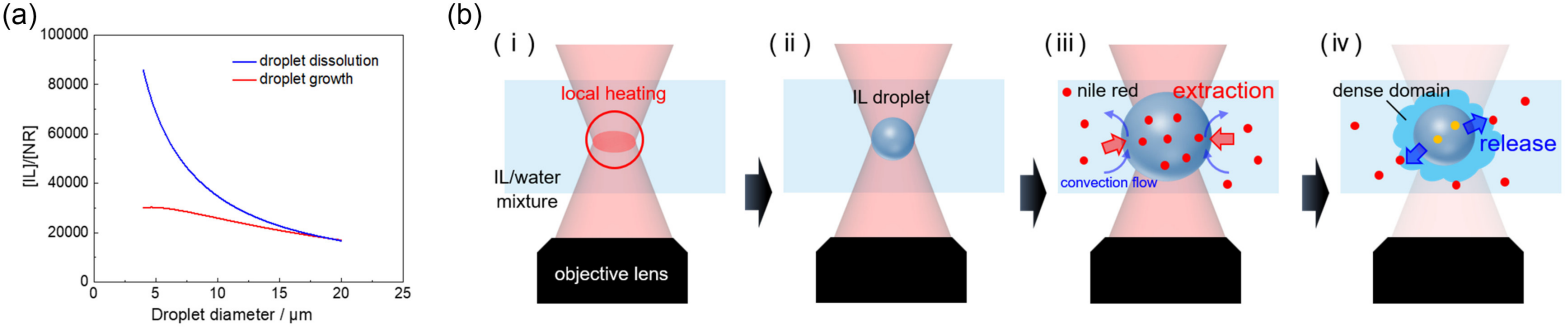
(a) The molar concentration ratio of the IL and NR within droplets in the growth and dissolution processes. (b) Schematic illustration of the molecular extraction/release dynamics in an IL microdroplet formed by optical tweezers.

Here, we consider the balance between IL and NR concentrations. We could not find density of [P_4448_]^+^[Br]^−^ from literatures, but it is reported that [P_4448_]^+^[Cl]^−^ has density of 0.94–0.96 g/mL in aqueous solutions (10–50 wt%) [29]. Based on this report, we here assume that the IL droplets consisting of [P_4448_]^+^[Br]^−^ have the constant density of 1.0 mg/mL for simplify, independent of *C*_IL_ and hence of droplet size. We can convert *C*_IL_ [wt%] to *C*_IL_ [mol/L] with this assumption. The IL molar concentration increases almost linearly with droplet size during the growth process, whereas it remains nearly constant and then decreases abruptly during droplet shrinkage (Figure S4). The NR molar concentration was estimated under the assumption that it is proportional to the integrated fluorescence intensity. Figure S5 shows the droplet‐size dependence of the NR concentration, which was derived from Figures 3b and 4b. The NR concentration exhibited an approximately linear relationship with the droplet diameter in both the growth and dissolution processes. Based on these trends, we calculated the molar concentration ratio between the IL and NR (Figure 5a). This ratio slightly decreased during the growth process. In contrast, the IL concentration relative to NR increased along with the droplet shrinkage. That is, NR molecules in a dissolving droplet can interact with a larger number of the IL compared to the growth process. As a result, the local environment around NR possibly becomes more hydrophobic within the shrinking droplet. Thus, the fluorescence color changes from red to orange, although *C*_IL_ decreases. Indeed, NR exhibits blue‐shifted fluorescence as its concentration decreases in the bulk aqueous IL solution (*C*_IL_ = 60 wt%) (Figure S6). Nonetheless, we are struggling to fully account for the results in the growth process (Figures 3c and 5a). We infer that the assumptions here may not sufficiently reflect the actual system.

Considering all the results above, we summarize molecular extraction/release dynamics of an IL single microdroplet under optical trapping conditions (Figure 5b). Upon focusing the NIR laser beam onto the IL solution, temperature elevation of ≈13 K is induced at the focal spot (Figure 5b(i)). This temperature rise was calculated on the basis of the literature [30]. The local solution temperature gets close to the critical point, triggering LLPS. Small droplets are gathered and fused to form a small single droplet in the optical potential; *U*_opt_,

$$U_{\mathrm{opt}}=\text{−}\frac{1}{2}\alpha_{\mathrm{pol}\;}{\left|E\right|}^{2}$$

where $\alpha_{\mathrm{pol}}$ is the polarizability of the solute and E is the electric field vector of the incident light [31]. Once an IL‐rich droplet is produced (Figure 5b(ii)), it promotes laser heating, because of the low thermal conductivity of the IL [32]. Accordingly, the temperature distribution becomes higher and wider. The former makes the droplet concentration increase. This is because, in the current IL/water mixture, the IL‐rich phase becomes denser with temperature elevation [8]. On the other hand, the wider temperature distribution induces LLPS outside the single droplet. The resultant condensates provide a source for the growth of the droplet trapped at the laser focus. Thus, *C*_IL_ increased in the growth process, accordingly extraction of NR is accelerated (Figure 5b(iii)). As previously reported [20], thermal convection or Marangoni convection is possibly caused by the local temperature gradient or the surface tension distribution at the droplet interface, respectively, during the droplet growth. The convection will enhance mass transport to the droplet (Figure 5b(iii)).

When *P*_eff_ decreases, the local temperature lowers, the convection is suppressed, and the droplet starts shrinking. We infer that the IL remains around the droplet during the dissolution process, because ILs have much small diffusion coefficients at high concentrations compared to typical solvents and ions [33, 34, 35]. Thus, it is expected that the solution surrounding the droplet is highly concentrated (Figure 5b(iv)). Due to this dense domain, *C*_IL_ is kept high even when the droplet shrinking proceeds. The existence of a dense domain also affects the concentration of NR within a droplet. The extraction efficiency strongly depends on the difference of IL concentrations between a droplet and the surrounding solution (Figure S7). In the dissolution process (20–8 µm in diameter), the concentration difference is possibly small due to the formation of a dense domain, leading to continuous release of NR from the droplet to the surrounding solution (Figure 5b(iv)). In the droplet, the NR concentration relative to *C*_IL_ decreases, exhibiting orange fluorescence. After the dense domain disappears, *C*_IL_ abruptly lowers. Accordingly, the local environment of NR becomes more hydrophilic, reverting red emission.

Finally, we discuss the extraction/release of NR from the viewpoint of the partition coefficient (*K*_d_). In the present experiments, the volume of the initial solution ($L_{\text{initial}}$) is ≈50 µL, whereas the volume of the IL droplets ($L_{\mathrm{IL}}$) is smaller than 10^−4^ µL. Because of $L_{\mathrm{IL}}$ ≪ $L_{\text{initial}}$, the concentration of NR in the water‐rich phase remains essentially unchanged from the initial concentration (${\left[\mathrm{NR}\right]}_{w}\approx {\left[\mathrm{NR}\right]}_{\text{initial}}$). Accordingly, $K_{d}$ can be approximated as $K_{d}\approx {\left[\mathrm{NR}\right]}_{\mathrm{IL}}/{\left[\mathrm{NR}\right]}_{\text{initial}}$, and the fluorescence enhancement of the IL droplets reflects the apparent value of $K_{d}$. Figure S8 shows the droplet‐size dependence of *K*_d_ calculated from Figure S5. It should be noted that, during the droplet dissolution process, the droplet is surrounded by a locally high‐concentration domain and the system is not under thermodynamically equilibrium. To further discuss how NR is concentrated in the IL droplets, we define the concentration factor (C) as $C={\left[\mathrm{NR}\right]}_{\mathrm{IL}}/{\left[\mathrm{NR}\right]}_{\text{initial}}$. The C can be expressed as

$$C=\frac{{\left[\mathrm{NR}\right]}_{\mathrm{IL}}}{{\left[\mathrm{NR}\right]}_{\text{initial}}}=\frac{K_{d}}{1+\left(K_{d}-1\right)\cdot \left(L_{\mathrm{IL}}/L_{\text{initial}}\right)}$$

This equation indicates that, if $K_{d}$ is constant, C increases as the droplet volume decreases. However, our experimental results show the opposite trend. This discrepancy suggests that $K_{d}$ is not constant but depends on droplet size (Figure S8), due to changes in the IL concentration within the droplets.

## 4 Conclusion

In this study, we investigated the molecular extraction and release dynamics of an optically controlled single IL microdroplet during its growth and dissolution processes, respectively. Raman microspectroscopy revealed that the IL concentration within the droplet monotonously increases with the growth, whereas it remains high for a while during dissolution. Fluorescence analysis showed that, during the dissolution process, the molecular release continuously proceeds owing to the formation of a dense domain surrounding the shrinking droplet. Moreover, we found that the local environment of the extracted molecules is strongly influenced by both the IL matrix and the molecular concentrations. These findings offer design principles for microanalytical techniques using optically controllable single microdroplets, as well as for applications involving the control of chemical reactions and photochemical processes in a small space.

## Funding

This work was supported Japan Society for the Promotion of Science (JP20H02550, JP22H05138, JP23H04600, JP25K08452); The CANON Foundation.

## Conflicts of Interest

The authors declare no conflicts of interest.

## Supporting information

The supporting information file contains fluorescence properties of NR in different solvents (Figure S1) and IL bulk solutions (Figures S2, S3 & S6), calculated IL and NR molar concentrations within droplets (Figures S4 & S5), the initial concentration dependence of the molecular extraction efficiency (Figure S7), and the partition coefficient of NR (Figure S8).
