# Supplementary material for: Molecular Extraction and Release of an Ionic Liquid Microdroplet With Dynamic Tunability Under Optical Trapping
Source: Chemphyschem. 2026 Jun 11;27(11):e70445. doi: 10.1002/cphc.70445 (PMC13261097; doi:10.1002/cphc.70445)
Supplement: Supplementary file 1 — The supporting information file contains fluorescence properties of NR in different solvents (Figure S1) and IL bulk solutions (Figures S2, S3 & S6), calculated IL and NR molar concentrations within droplets (Figures S4 & S5), the initial concentration dependence of the molecular extraction efficiency (Figure S7), and the partition coefficient of NR (Figure S8). [file CPHC-27-e70445-s001.pdf]

Supporting Information for

Molecular Extraction and Release of an Ionic Liquid Microdroplet with Dynamic Tunability under Optical Trapping

Kosuke Nakatsu,[a] Rai Kobayashi,[a] Moe Akazawa,[a] Yasuyuki Tsuboi,[a] and Ken-ichi Yuyama*[a]

E-mail: k-yuyama@omu.ac.jp

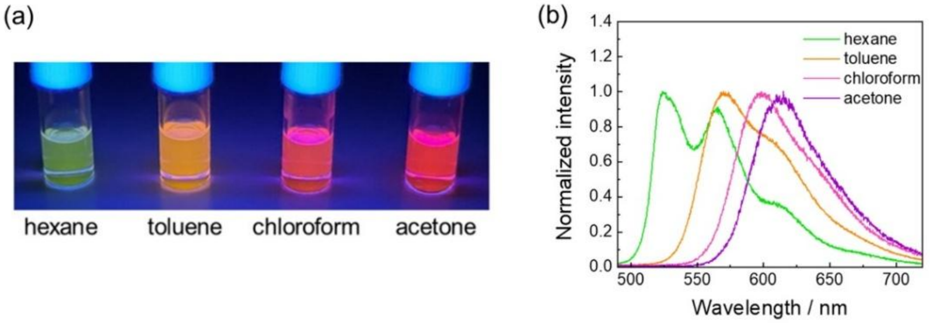

Figure S1. Fluorescence (a) images and (b) spectra of NR in different solvents

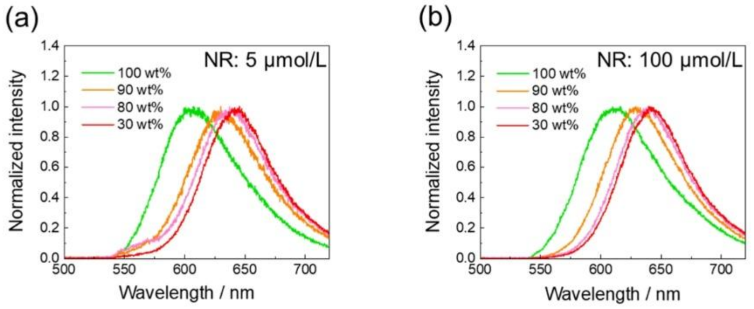

Figure S2. Fluorescence spectra of IL bulk solutions (C_{IL}: 30–100 wt%) containing NR at concentrations of (a) 5 μmol/L and (b) 100 μmol/L

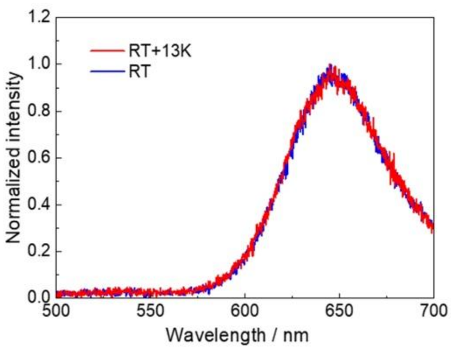

Figure S3. Temperature dependence of fluorescence spectra of NR (5.0 μmol/L) in the IL bulk solution (C_{IL}: 5.0 wt%)

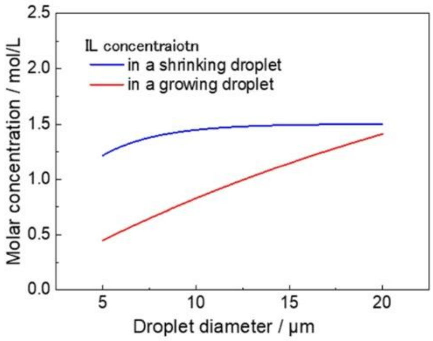

Figure S4. The IL molar concentration within droplets during growth and dissolution

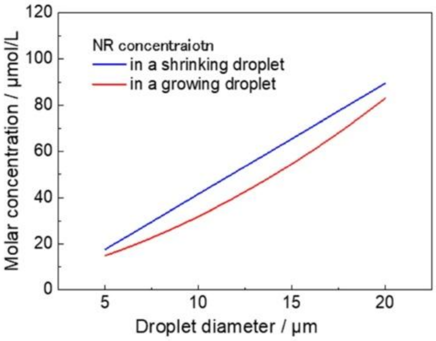

Figure S5. The NR molar concentration within droplets during growth and dissolution

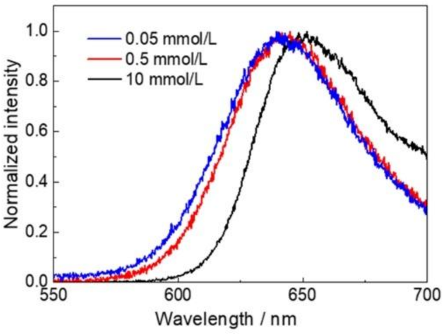

Figure S6. Fluorescence spectra of NR with different concentrations in an IL bulk solution (C_{IL}: 60 wt%)

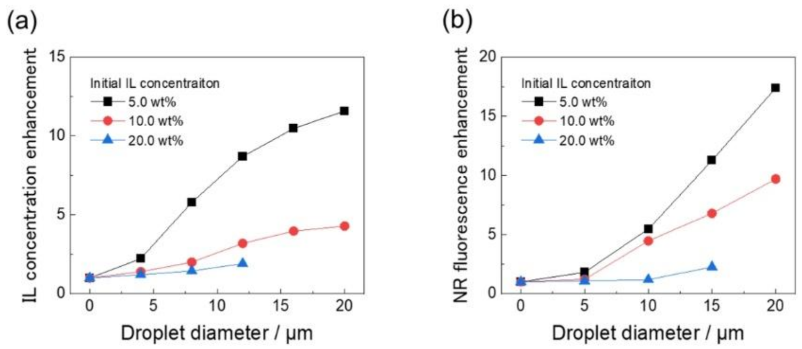

Figure S7. (a) The IL concentration enhancement of droplets preared from 5, 10, and 20 wt% IL solutions (b) The fluorescence enhancement of NR extracted to droplets of the panel (a)

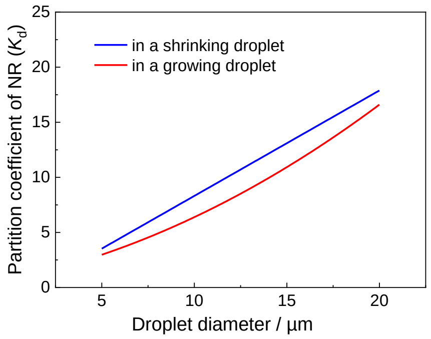

Figure S8. The partition coefficient (K_d) of NR calculated from Fig. S5
